# Identification of virus-rich intermediate cells as crucial players in SARS-CoV-2 infection and differentiation dynamics of human airway epithelium

**DOI:** 10.3389/fmicb.2024.1507852

**Published:** 2024-12-13

**Authors:** Mi Il Kim, Choongho Lee

**Affiliations:** College of Pharmacy, Dongguk University, Seoul, Republic of Korea

**Keywords:** SARS-CoV-2, airway epithelial cell, air-liquid interface culture, single-cell RNA sequencing, virus-rich intermediate

## Abstract

Understanding the early interactions between severe acute respiratory syndrome coronavirus 2 (SARS-CoV-2) and human airway epithelial cells is essential for unraveling viral replication and spread mechanisms. In this study, we investigated the early dynamics of airway epithelial cells during SARS-CoV-2 infection using well-differentiated human nasal and tracheal epithelial cell cultures by incorporating three publicly available single-cell RNA sequencing datasets. We identified a previously uncharacterized cell population, termed virus-rich intermediate (VRI) cells, representing an intermediate differentiation stage between basal and ciliated cells. These VRI cells exhibited high viral loads at all infection time points, strong interferon and inflammatory responses, increased mRNA expression of microvilli-related genes (PAK1, PAK4, VIL1), and suppression of apoptosis markers (BAX, CASP3) alongside increased anti-apoptotic gene expression (BCL2). Cell-cell interaction analysis revealed that VRI cells send signals to basal cells via receptor-ligand pathways such as EPHA and VEGF, likely promoting basal cell differentiation and proliferation through MAPK signaling. These findings suggest that SARS-CoV-2 utilizes VRI cells as a primary site for replication and spread, leveraging these cells’ unique differentiation state to evade host cell death and facilitate viral propagation. This study provides insights into the early cellular responses to SARS-CoV-2 infection and highlights potential therapeutic targets to limit viral spread.

## 1 Introduction

Severe acute respiratory syndrome coronavirus 2 (SARS-CoV-2), first reported in Wuhan, Hubei Province, China in December 2019, rapidly spread worldwide, leading the World Health Organization (WHO) to declare a pandemic on March 11, 2020 (Huang et al., 2020; Adil et al., 2021). The pandemic has resulted in millions of deaths globally and has had a profound impact on economic, social, and healthcare systems (Adil et al., 2021). SARS-CoV-2 shares similarities with previously emerged coronaviruses, such as severe acute respiratory syndrome coronavirus 1 (SARS-CoV-1) and middle eastern respiratory syndrome coronavirus (MERS-CoV) (Rabaan et al., 2020), particularly in its unique mechanism of infection involving the binding to ACE2 receptors to invade human cells (Perrotta et al., 2020). The structure and mutations of the spike protein play a critical role in the virus’s transmissibility and pathogenicity, with numerous variants such as alpha, beta, delta, and omicron having emerged (Mohammadi et al., 2021; Ullrich et al., 2022). According to recent WHO reports, variants like KP.3 and LB.1 are showing an increasing prevalence globally. Although the number of new cases and deaths has decreased, the positivity rate is rising again (Emerging Diseases and Zoonoses (EZD), EPP, 2024).

To better understand the biology of SARS-CoV-2, various studies have been conducted using patient samples. However, these studies often involve testing several days after SARS-CoV-2 infection, making it difficult to understand the initial interactions between the respiratory epithelial cells and the virus (Ziegler et al., 2021; Tian et al., 2022). Understanding these early interactions is crucial for the development of vaccines and therapeutics. To overcome this challenge, several research have been conducted on early virus infection using *in vitro* models, specifically human airway epithelial cells cultured at the air-liquid interface (ALI) (Silva et al., 2023; Bukowy-Bieryllo et al., 2022). These *in vitro* models, with their potential to promote cell differentiation and optimize airway epithelial cells’ morphological and histological characteristics, have been used frequently to study the effects of SARS-CoV-2 infection on host cells (Fiege et al., 2021; Gamage et al., 2022; Hatton et al., 2021; Ravindra et al., 2021). In addition, recent studies have shown that applying single-cell RNA sequencing technology provides information on differentiation pathways for various cell types and cellular responses to viral infection (Fiege et al., 2021; Gamage et al., 2022; Ravindra et al., 2021).

To gain an insight on the early events associated with host cell infection by SARS-CoV-2, we performed the in-depth reanalysis of the datasets from three published papers that investigated the immune, entry factors, and cellular state changes in airway epithelial cells in response to SARS-CoV-2 infection using the ALI model (Fiege et al., 2021; Gamage et al., 2022; Ravindra et al., 2021). Some studies indicate that the target cells of SARS-CoV-2 are ciliated cells (Fiege et al., 2021; Ravindra et al., 2021), while others suggest that most cell types are infectable with SARS-CoV-2 (Gamage et al., 2022). The studies also explain the immune response to viral infection, including the induction of interferon (IFN)-*α* and IFN-*γ* and an increase in interleukin (IL)-6 (Fiege et al., 2021; Gamage et al., 2022; Hatton et al., 2021; Ravindra et al., 2021). Most studies have focused on ciliated cells, known as target cells of SARS-CoV-2, and the immune response to viral infection. When we analyzed three integrated data sets, we were able to identify cell populations characterized by the heavy accumulation of viral genes. We found these cells are intermediate cells transitioning from basal to ciliated cells. However, no studies have yet to elucidate the molecular features of this cell population distinct from rest of cells. Therefore, we investigated the properties of this unique cell population to better understand how SARS-CoV-2 utilizes it in its life cycle and how airway epithelial cells respond to the infection.

## 2 Materials and methods

### 2.1 Data collection

We used three publicly available single-cell RNA sequencing (scRNA-seq) datasets: GSE166766 (Illumina NovaSeq 6000) (Ravindra et al., 2021), GSE157526 (Illumina NovaSeq 6000) (Fiege et al., 2021), and GSE182475 (Illumina HiSeq 4000) (Gamage et al., 2022). The datasets consist of human epithelial cells infected with SARS-CoV-2 under varying conditions, including different time points post-infection. Raw sequencing data were downloaded from the NCBI Gene Expression Omnibus (GEO) and processed using the Seurat pipeline for quality control, normalization, and integration (Hao et al., 2024).

### 2.2 Data processing

The three single-cell RNA sequencing (scRNA-seq) datasets were pre-processed using the Seurat R package (v 5.0.3) (Hao et al., 2024). Quality control was conducted by filtering out cells that exhibited over 30% mitochondrial content, as this may indicate low-quality or damaged cells. Additionally, cells with fewer than 200 detected genes were removed from the analysis to ensure robust representation of the cellular transcriptome. Similarly, features (genes) detected in fewer than three cells were excluded. To mitigate the potential bias caused by variability in cell numbers across datasets and time points, we employed a subsampling strategy (Seirup et al., 2020). The subsample size was determined by the smallest number of cells present in any dataset, which was 1,700 cells. Therefore, 1,700 cells were randomly selected from each time point to ensure a balanced and comparable analysis across all datasets. For the 28-day post-infection (dpi) time point, present only in dataset GSE166766, a larger number of cells was available. To maintain consistency in the total number of cells analyzed across time points, we subsampled 3,400 cells for this time point. The subsampling was implemented using the dplyr R package (v1.1.4), ensuring uniformity across datasets while minimizing potential skew due to unequal cell representation.

The subsampled datasets were integrated using the Seurat R package (v 5.0.3) to eliminate batch effects and create a unified dataset for comparative analysis (Hao et al., 2024). The integration was performed following the standard Seurat v5 workflow, employing the anchor-based canonical correlation analysis (CCA) method via the “IntegrateLayers” function. This method aligns similar cell populations across datasets by identifying anchors, or common cellular states, between samples, thereby effectively mitigating technical variation.

### 2.3 Clustering and cell type annotation

For visualization, uniform manifold approximation and projection (UMAP) was performed on the top 20 principal components. Clustering analysis was conducted on the principal components analysis (PCA)-reduced data using Seurat (v 5.0.3) with a resolution parameter set to 0.8 to derive the UMAP representation. Specific marker genes were employed to identify and annotate each cell type, selected based on an extensive review of the literature and previously published single-cell RNA sequencing studies (Fiege et al., 2021; Gamage et al., 2022; Hatton et al., 2021; Ravindra et al., 2021; Hewitt and Lloyd, 2021). These cell types included basal cells, ciliated cells, secretory cells, and other airway epithelial cell populations. The primary marker genes used for cell type identification are detailed in the Supplementary Table S2.

### 2.4 Trajectory analysis

To infer the differentiation trajectory of airway epithelial cells and identify the stages of VRI cells development, we conducted pseudo-time analysis using the Monocle3 R package (version 1.3.5) (Trapnell et al., 2014; Qiu et al., 2017). Pseudo-time was assigned to each cell using the order_cells function, which positions cells along the inferred trajectory based on their transcriptional similarity and progression. The starting root and branching points of the trajectory were manually annotated using known biological insights and the spatial distribution of the identified cell types. The calculated pseudo-time scores were then incorporated into the metadata of the respective Seurat objects to be visualized on existing UMAP embeddings.

### 2.5 Gene ontology analysis

Gene ontological analysis was performed using the “clusterProfiler” package (v 4.6.2) to identify significantly over-represented biological process (BP) GO terms among differentially expressed genes (DEGs) (Wu et al., 2021; Yu et al., 2012). DEGs were determined with the FindAllMarkers function, applying thresholds of min.pct = 0.25 and logfc.threshold = 0.25, across various conditions. These conditions included different VRI cells subtypes to distinguish functional differences between clusters, as well as the bulk population at different time points to capture global trends during infection. We focused on DEGs with an adjusted *p*-value < 0.05, using the org.Hs.eg.db database for *Homo sapiens*. GO enrichment analysis was similarly filtered with an adjusted *p*-value < 0.05. The resulting GO terms were carefully curated to remove redundant or irrelevant entries, and the most significant terms were visualized based on gene ratio. The genes linked to these GO terms were further used in various downstream analyses, including plotting and scoring.

### 2.6 Cell-cell interaction analysis

Cell-cell interaction analysis was performed using the CellChat package (v 1.6.1) to examine the signaling interactions between VRI cells and other cell types at various time points post-infection (Jin et al., 2021). The data were divided by time points to assess changes in cell-cell communication for the infection. Default plotting functions from CellChat, including the netVisual_bubble function, were utilized to visualize these interactions. The netVisual_bubble plots were generated with a default *p*-value threshold of 0.05 to determine significant interactions.

## 3 Results

### 3.1 Integration and analysis of scRNA-seq data from SARS-CoV-2-infected ALI cultures experiments

To gain an insight on overlapping common effects of coronavirus infection on transcriptional profiles of different host cell populations in a single-cell resolution, we reanalyzed scRNA-seq data from air-liquid interface (ALI) cultures of nasal and bronchial cells infected with SARS-CoV-2. This analysis integrated datasets from 3 previously published studies (Fiege et al., 2021; Gamage et al., 2022; Ravindra et al., 2021) (Table 1), encompassing 10 samples and 99,775 cells across five infection time points (mock, 1 day post-infection (dpi), 2 dpi, 3 dpi, and 28 dpi). Due to significant variability in the number of cells sequenced in each study and at different time points, we normalized the datasets by subsampling 1,700 cells from each time point within each study. For the 28 dpi time point, a subsampling of 3,400 cells was executed to align the total cell count with other time points, as this time point exclusively exists in the data from Gamage et al. (2022) (Supplementary Table S1). This subsampling ensured a balanced, integrated dataset and mitigated potential biases from cell count discrepancies (Seirup et al., 2020). The integration of these uniformly subsampled datasets enabled a robust comparative analysis across the different conditions and time points represented.

**Table 1:** Summary of scRNA-seq datasets used in the study.

Papers	Accession	ALI culture cell	Infection time (dpi)	Number of cells (original)	Number of cells (subsampled)
Ravindra et al. (2021)	GSE166766	Bronchial epithelial cells	Mock, 1, 2, 3	77,143	6,800
Fiege et al. (2021)	GSE157526	Tracheal bronchial epithelial cells	Mock, 1, 2	8,646	5,100
Gamage et al. (2022)	GSE182475	Nasal epithelial cell	Mock, 3, 28	13,986	6,800

**Figure 1:**
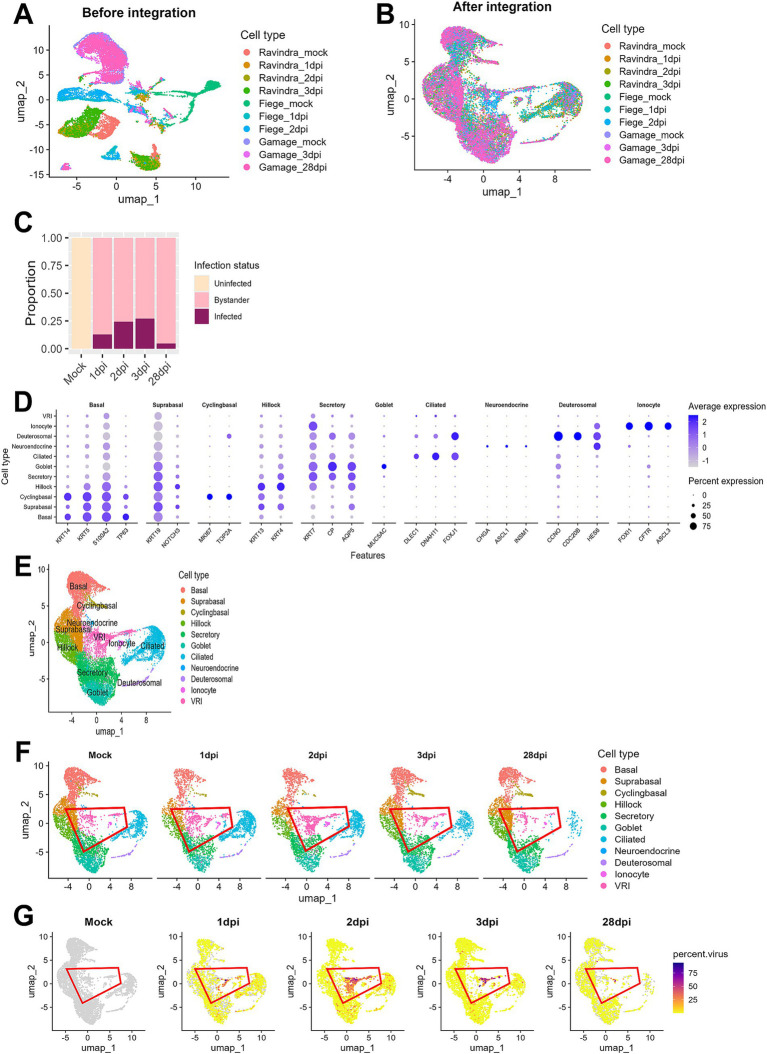
Integration and analysis of scRNA-seq data from SARS-CoV-2-infected ALI cultures. **(A)** UMAP plot displaying scRNA-seq data before integration, showing separate clustering of individual datasets by sequencing platform or sample batch; **(B)** UMAP plot of scRNA-seq data after batch correction and integration using Seurat, showing harmonized cell clustering across all datasets. **(C)** Bar plot illustrating the proportion of cells across different infection time points after integration. **(D)** Bar plot displaying the proportion of cells at days post-infection. Cells were classified into three groups: uninfected (Mock), infected (cells with percent viral gene >0.1%), and bystander (cells not infected but exposed to the infection environment); **(E)** UMAP plot showing annotated cell clusters after integration, labeled according to the cell types; **(F)** UMAP plots showing temporal changes in cell population composition across days post-infection. Each color represents the different cell types, and the red outline highlights VRI stage cells; **(G)** UMAP plots show the distribution of infected cells (percent viral load > 0.1%) across the time points. Viral load is represented by a color scale, with warmer colors indicating higher viral load.

A clear batch effect was observed upon initial visualization of the combined data (Figure 1A). However, after integrating the data using the Seurat method (Hao et al., 2024), the cells from different studies clustered together based on their biological characteristics, indicating that batch effects were effectively eliminated (Figure 1B). The ALI cultures were originally composed of uninfected (mock) samples and samples infected with SARS-CoV-2 at 1, 2, 3, and 28 dpi. Cells in the mock samples were defined as uninfected. In the samples treated with SARS-CoV-2, we identified cells as infected if they had viral reads exceeding 0.1% of total RNAs per cell and as bystander cells, if they were exposed to the virus but failed to satisfy this condition. This approach effectively controlled background noise from misaligned reads in the mock samples. In the analysis of viral infection progression over time, it was noted that the number of infected cells peaked at 3 dpi and then declined by 28 dpi, indicating a reduction in virus-infected cells over the long period of time (Figure 1C).

When we conducted cell typing using the top DEGs and known cell markers, we were able to identify 10 major cell populations. They include basal, suprabasal, cyclingbasal, hillock, secretory, goblet, ciliated, neuroendocrine, deuterosomal, and ionocyte cells (Figures 1D,E; Supplementary Table S2). Interestingly, we discovered a unique cell cluster that did not correspond to any known cell markers. Due to their intermediate differentiation status progressing from basal to ciliated cells (Figure 1F) and viral transcripts-enriched characteristics (Figure 1G) throughout all time points, we decided to name this cell population as virus-rich intermediates (VRI) cells. When examining the proportion of infected cells across different cell types, we found that this cluster had a notably high percentage of virus-infected cells (>50%) (Supplementary Figure S1). Consequently, we focused our subsequent analyses on these VRI cells to further elucidate their potential role in SARS-CoV-2 infection.

### 3.2 Molecular characterization of three subclusters of the VRI cells

**Figure 2:**
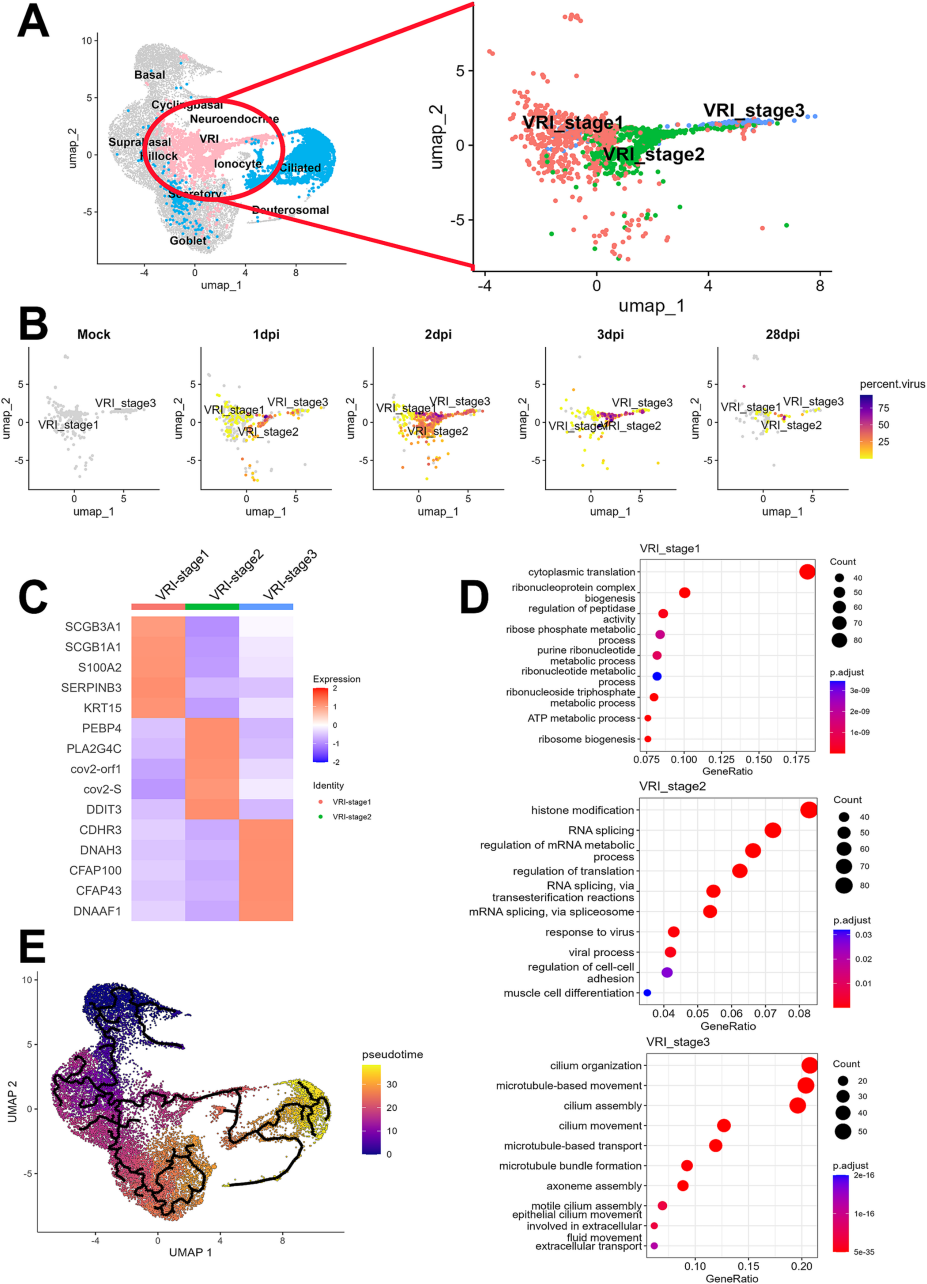
Elucidation of VRI as a cell population differentiating from basal to ciliated cells. **(A)** UMAP projection of the full dataset on the left and a sub-clustering of VRI-stage clusters (VRI_stage1, VRI_stage2, and VRI_stage3) on the right; **(B)** UMAP plots displaying viral load distributions across time points. Cells are colored by viral load percentage, illustrating dynamic changes within VRI-stage clusters; **(C)** Heatmap showing the expression levels of key marker genes across VRI_stage1, VRI_stage2, VRI_stage3; **(D)** Dot plots representing the top enriched pathways and biological processes in VRI_stage1, VRI_stage2, and VRI_stage3 clusters. Dot size corresponds to the gene ratio, and color intensity indicates the adjusted *p*-value for pathway enrichment; **(E)** Pseudo-time trajectory analysis performed using Monocle3, illustrating the differentiation pathways of cells. Cells are color-coded by pseudo-time.

To further investigate the newly identified VRI cells, we performed the detailed analysis of this VRI cells cluster to identify subclusters. Based on differential gene expression patterns, we found three VRI cells subtypes, which we arbitrarily named VRI_stage1, VRI_stage2, and VRI_stage3 (Figure 2A). VRI_stage1 was characterized by high expression of basal markers (S100A2 and KRT15) and secretory markers (SCGB1A1 and SCGB3A1) and the stress-response gene SERPINB3, which is known to be upregulated in response to cellular stress (Figure 2C). It was identified as early-stage cells differentiating from basal to ciliated. VRI_stage2, a newly generated and maintained population from 1 dpi through 28 dpi (Figure 2B), showed increased expression of SARS-CoV-2 gene (cov2-orf1 and cov2-S) and lipid metabolism-related genes (PEBP4, PLA2G4C, and DDIT3) (Figure 2C). We defined it as newly infected cells. Lastly, VRI_stage3 showed increased expression of cilium-related genes including CDHR3, DNAH3, SFAP100, CFAP43, and DNAAF1 (Figure 2C). It was identified as pre-ciliated cells just before fully differentiating into ciliated cells (Figure 2B).

The analysis of DEGs among the subtypes of VRI cells and subsequent GO enrichment analysis revealed the potential roles of VRI cells during SARS-CoV-2 infection across each subtype (Figure 2D). In VRI_stage1, GO terms related to cytoplasmic translation, ribonucleoprotein complex assembly, ribosome biogenesis, and various metabolic processes were prominent. This indicates that VRI_stage1 cells are in a state of high metabolic and biosynthetic activity. In VRI_stage2, active responses to viral infection were characterized by enriched GO terms, including histone modification, RNA splicing, and viral processes. Notable pathways related to RNA splicing via transesterification reactions and spliceosome-mediated splicing were observed, suggesting a key role in gene regulation and adaptation to viral infection. Finally, VRI_stage3 predominantly featured GO terms related to cilium organization and assembly, including microtubule and axoneme assembly. This suggests that VRI_stage3 cells are heavily involved in cilia formation and function.

When we conducted trajectory analysis to determine the timing of the appearance of these VRI cells during the differentiation of nasal and tracheal epithelial cells, we found that VRI cells are situated between basal and ciliated cells (Figure 2E). As expected, pseudo-time progression suggested the following this sequence of differentiation: VRI_stage1, VRI_stage2, VRI_stage3, and ciliated cells (Supplementary Figure S2).

### 3.3 VRI cells serves as a primary site for SARS-CoV-2 infection

**Figure 3:**
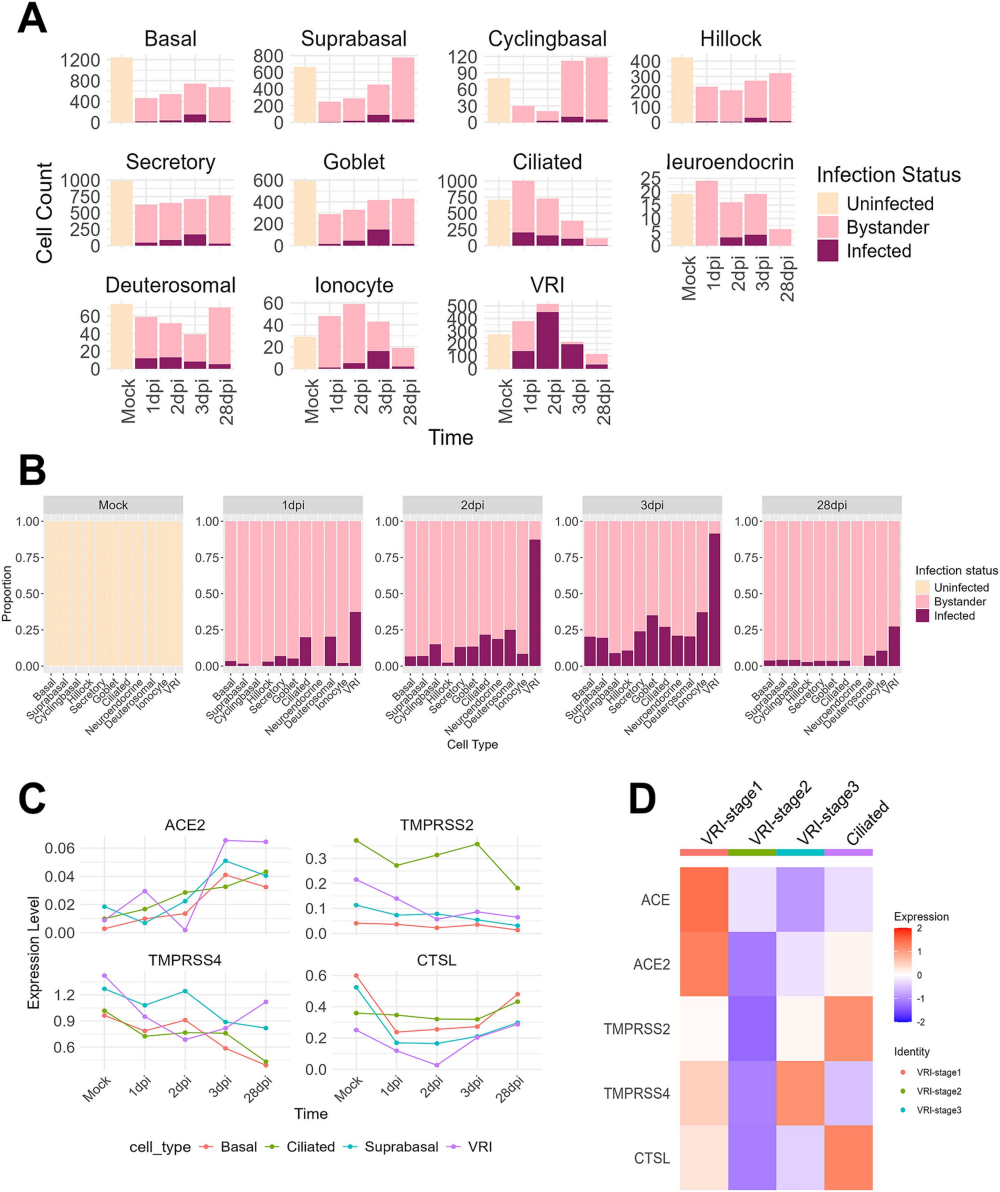
VRI serves as a primary site for SARS-CoV-2 infection. **(A)** Bar plots showing the cell counts of infected, uninfected, and bystander cells for different cell types across time points; **(B)** Bar plots depicting the proportions of infected, uninfected, and bystander cells for different cell types across time points; **(C)** Line plots representing the expression levels (log-normalized values) of viral entry and response genes (e.g., ACE2, TMPRSS2, CTSL) over time across different cell types; **(D)** Heatmap showing the expression of viral entry genes (ACE2, TMPRSS2, CTSL, etc.) across VRI-stage cells and ciliated cells relevant populations at time points. The color scale indicates *Z* score-transformed expression level. Infected cells are shown in purple, bystander cells in light pink, and uninfected cells in gray.

To investigate the relationship between VRI cells and SARS-CoV-2 infection, we first examined the number and proportion of infected cells by cell type. It is well established that ciliated cells are the primary target cells for SARS-CoV-2 (Fiege et al., 2021; Ahn et al., 2021; Sano et al., 2022; Robinot et al., 2021; Sims et al., 2005). At 1 dpi, while the number of infected cells in ciliated cells sharply increases, VRI cells also show a similar number of infected cells to ciliated cells. However, at 2 dpi and 3 dpi, while the number of infected ciliated cells decreases, both the proportion and number of infected cells in VRI increases (Figures 3A,B). Notably, VRI cells exhibit the highest number of infected cells at 2 dpi, surpassing all other cell types. This elevated proportion of infected cells in VRI cells remains higher than other cell types, even at 28 dpi (Figure 3B). To first understand why the number and proportion of infected cells in VRI cells are higher than in other cell types, we examined the expression of known SARS-CoV-2 host factor genes including ACE2, TMPRSS2, TMPRSS4, and CTSL. ACE2 expression was similar between VRI and ciliated cells at the mock stage but increased more rapidly in VRI cells as the infection progressed (Figure 3C). Although TMPRSS2 expression was higher in ciliated cells, it was also significantly expressed in VRI cells, suggesting that it could facilitate viral entry in these cells.

Interestingly, in VRI_stage2, the most heavily infected stage by the virus within VRI cells, there was almost no expression of genes related to SARS-CoV-2 entry (Figures 3C,D). This result aligns with previous studies that have shown downregulation of ACE2 expression following SARS-CoV-2 infection (Lu et al., 2022). In contrast, VRI_stage1, which precedes VRI_stage2 in the pseudo-time developmental trajectory (Figure 2E; Supplementary Figure S2), exhibited higher expression of entry-related genes, indicating that VRI_stage1 likely serves as the primary entry point for SARS-CoV2. VRI cells exhibit strong interferon and inflammatory responses.

**Figure 4:**
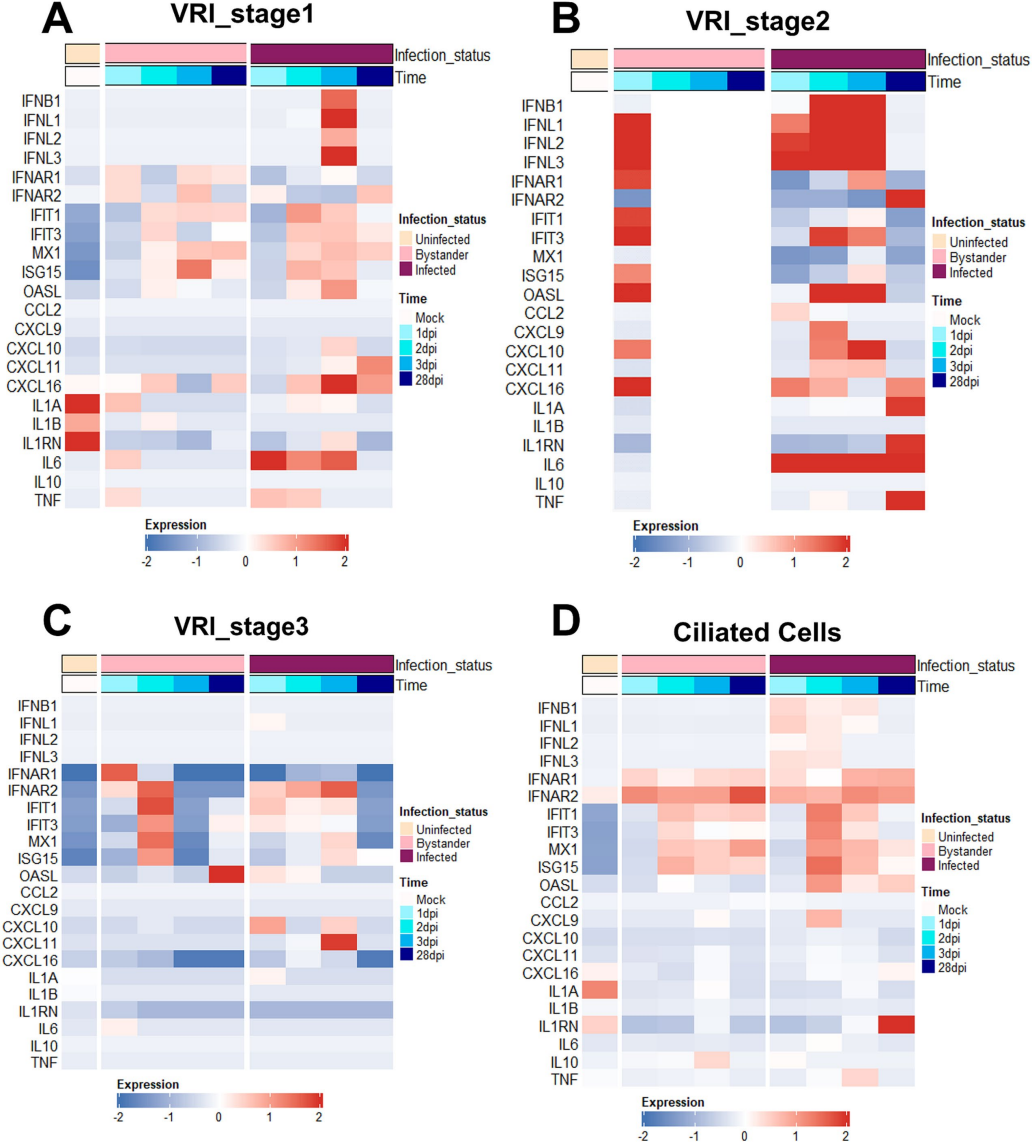
VRI cells exhibit strong interferon and inflammatory responses. **(A–D)** Heatmaps depicting the expression levels of key immune response genes across time and infection status in different cell populations; **(A)** VRI_stage1 cells; **(B)** VRI_stage2 cells; **(C)** VRI_stage3 cells; **(D)** Ciliated cells; Each column represents a pseudobulk average of all cells from a single cell type and single study. The color scale indicates z-score-transformed expression levels, with red representing higher expression and blue representing lower expression.

We investigated genes related to interferon and inflammatory responses to understand how immune responses differ after infection between VRI and other cell types. Compared with other stages of cells, a much stronger interferon and inflammatory response was observed in VRI_stage2 upon infection. Both infected and bystander cells of VRI_stage2 exhibited strong immune responses (Figure 4B). The absence of expression in the uninfected and parts of the bystander columns (white spaces) reflects the fact that VRI_stage2 cells only emerge after infection, meaning there are no uninfected cells in VRI_stage2. Furthermore, from 2 dpi, all cells in VRI_stage2 are infected, which explains the lack of representation for bystander cells at later time points. IFNL1, an interferon-λ gene, was highly expressed as early as 1 dpi, while IFNB1 expression peaked later (Figure 4B). ISGs such as OAS1, MX1, and ISG15 were also activated, and the pro-inflammatory chemokine CXCL10 was significantly upregulated in both infected and bystander cells of VRI_stage2 (Figure 4B). These results suggest that VRI_stage2 is highly sensitive to SARS-CoV-2 infection and plays a critical role in mounting a strong immune response. Interestingly, in VRI_stage2, the expression of certain ISGs, including IFIT1 and ISG15, was relatively low (Figure 4B). Despite VRI_stage2 being the cell group with the highest levels of SARS-CoV-2 transcripts, these genes were less activated compared to VRI-stage1 (Figure 4A). This suggests a possible viral immune evasion mechanism that suppresses the activation of these specific ISGs.

In VRI_stage1, the expression of IFNL1 and IFNB1 was initially low but increased over time, with a delayed yet eventual activation of IFIT1 and ISG15 from 2 dpi (Figure 4A). This suggests that these cells can still mount an antiviral response but react more gradually. This implies that VRI_stage1 cells become more involved in the immune response as the infection progresses, potentially compensating for the reduced ISG response in VRI_stage2. In contrast, VRI_stage3 showed little to no expression of IFNL1 and IFNB1, with reduced ISG levels (Figure 4C), indicating that these cells may be transitioning into differentiation and may be less involved in direct antiviral defense at this stage. In ciliated cells, IFNL expression was observed from 1 dpi, but at lower levels than in VRI_stage2, and ISG expression was moderate (Figure 4D). This supports the understanding that their capacity to mount a strong interferon response is limited, often leading to cell damage due to viral infection rather than effective immune defense.

Compared to VRI, other cell types, such as basal cells, showed relatively low expression of IFNB and IFNL. However, ISGs such as IFIT1, IFIT3, and MX1 were highly expressed in basal cells (Supplementary Figure S3A). This suggests that even though basal cells do not produce large amounts of interferon, they may have been stimulated by interferon from other cells. In contrast, suprabasal cells exhibited little to no ISG expression (Supplementary Figure S3B), suggesting that these cells may be more involved in tissue repair or other functional roles rather than in inflammatory responses. A relatively strong response was observed in secretory cells, with high expression of ISGs such as IFIT1, IFIT3, and MX1, along with the chemokines CXCL9, CXCL10, and CXCL11 (Supplementary Figure S3C). This suggests that secretory cells provide physical defense through mucus during viral infection and play a role in recruiting immune cells to the site of infection through chemokines.

Moreover, IL1A and IL1B showed minimal changes across all cell types (Figure 4; Supplementary Figure S3), consistent with prior studies indicating that IL1 does not play a significant role in early airway immune responses to SARS-CoV-2 infection (Ravindra et al., 2021; Barnett et al., 2023). However, at 28 dpi in VRI-stage2, IL-1A is highly expressed. IL6 and TNF were significantly upregulated in VRI_stage1 and VRI_stage2, contributing to the inflammatory response, and it was observed that they were upregulated in both basal and secretory cells at 28 dpi, which is a considerable time after infection (Figure 4B). This result is similar to increased IL-6 in patients with SARS-CoV-2 infection and increased IL-1 in severe patients (Fodor et al., 2022; Lucas et al., 2020). This suggests that it could be due to a persistent immune response to the remaining virus in the VRI stage or chronic inflammation. Thus, VRI cells exhibit higher immune gene expression levels than other cell types, demonstrating their important role in the defense mechanism against SARS-CoV-2 infection.

### 3.4 SARS-CoV-2 induced changes in epithelial cell composition

**Figure 5:**
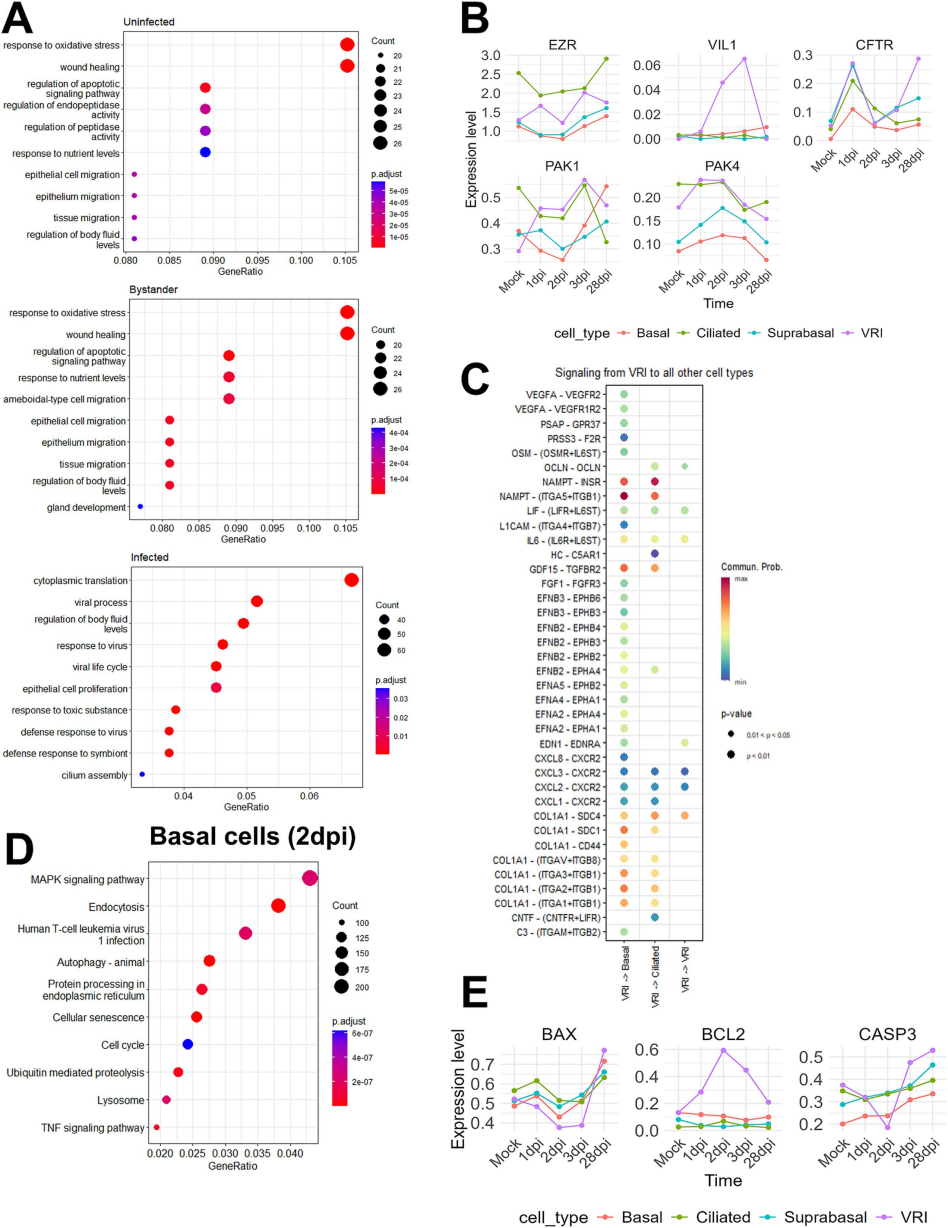
SARS-CoV-2 induced changes in epithelial cell composition. (A) Dot plots showing the top enriched pathways and biological processes in VRI-stage cells, divided into three groups: uninfected, infected, and bystander cells. The dot size represents the number of genes involved in each pathway, and the color scale indicates adjusted *p*-values; (B) Line plots showing the temporal expression dynamics of markers related to microvilli formation (e.g., EGFR, IL6, and VEGFA) in various cell types. The expression values represent log-normalized pseudobulk data obtained using Seurat’s “AggregateExpression” function; (C) Bubble plot displaying the cell-cell interaction signaling analyzed using CellChat, showing signaling sent from VRI-stage cells to other cell types at 2dpi. The color scale represents the communication probability (Comm. Prob.), and the size of the bubbles indicates p-values for the identified interactions; (D) Dot plot illustrating the top GO enrichment pathways in basal cells at 2dpi. The dot size represents the number of genes involved, and the color scale indicates adjusted p-values; (E) Line plots showing the temporal expression dynamics of markers related to apoptosis across various cell types over time. The expression values represent log-normalized pseudobulk data obtained using Seurat’s “AggregateExpression” function. Lines represent the average gene expression level in each cell type at different time points post-infection.

Our research aims to uncover the reasons behind the persistence of SARS-CoV-2 infection in VRI cells and the significantly higher amounts of viral transcripts. To investigate why there is an accumulation of viral transcripts in VRI cells post-infection, we first conducted GO enrichment analysis for uninfected, bystander, and infected cells between non-VRI and VRI cells. Our GO enrichment analysis revealed that bystander and uninfected cells showed similar enrichment in pathways such as wound healing, epithelial cell migration, and tissue migration. However, bystander cells exhibited additional enrichment in ameboidal-type cell migration and gland development, suggesting a role in tissue remodeling and immune responses. In infected cells, significant enrichment was found in viral processes, regulation of body fluid levels, and cilium assembly, highlighting their involvement in both viral replication and airway fluid regulation (Figure 5A). Interestingly, the GO analysis of VRI_stage3 revealed a strong enrichment for cilium-related processes, including cilium organization and microtubule-based movement (Figure 2D). This suggests that as infected cells progress into VRI_stage3, they initiate processes related to cilia formation.

Building on the foundation Chien-Ting Wu et al. established, our study further elucidates the complex mechanisms of SARS-CoV-2 infection. Their research demonstrated that SARS-CoV-2 enters cells via cilia and promotes viral production through microvilli formation (Wu et al., 2023). By examining the expression levels of microvilli marker genes across various cell types, we found that VRI cells show increased expression of EZR, a key gene for microvilli formation, and VIL1, a structural protein of microvilli (Figure 5B). Additionally, the PAK1 and PAK4 kinases, identified by Chien-Ting Wu et al. as regulators of microvilli dynamics, were upregulated. This suggests that, after infecting VRI cells, SARS-CoV-2 optimizes them by inducing the formation of both cilia and microvilli, facilitating viral entry and release.

To investigate post-infection changes in cell populations, including the increase in VRI cells and the decrease in ciliated and basal cells, we utilized CellChat to explore ligand-receptor interactions (Jin et al., 2021). Since VRI cells are heavily infected by SARS-CoV-2 and likely play a crucial role in the virus’s life cycle, we focused on the signals these cells send to other cell types. On 2 dpi, VRI cells were found to send NAMPT and COL1A1 signals to both basal and ciliated cells with the highest communication probabilities (Figure 5C). Although the NAMPT and COL1A1 signaling pathways exhibited high communication probabilities targeting both basal and ciliated cells, we also identified signals specifically directed toward basal cells that are of particular interest despite having lower communication probabilities. These signals included EPHA, EPHB, and VEGF, which were found to be transmitted from VRI cells to basal cells (Figure 5C). Both signaling pathways commonly involve the MAPK pathway downstream (Jiang et al., 2024; Wilkinson, 2014; Perez White and Getsios, 2014; Doanes et al., 1999). Upon performing GO enrichment analysis for basal cells on 2 dpi, we observed that the MAPK signaling pathway was highly enriched (Figure 5D). This suggests that, following SARS-CoV-2 infection, signals such as EPHA, EPHB, and VEGFA are transmitted from VRI cells to basal cells, promoting the differentiation and growth of the basal cells. This finding is crucial in understanding the temporal changes observed in the UMAP plot, where basal cell numbers decrease at 1 dpi and return to normal levels by 3 dpi (Figure 1F). Additionally, while VRI and ciliated cells increase at 1 dpi, only VRI cells significantly increase at 2 dpi (Figure 3A). This indicates that basal cell differentiation contributes to the expansion of VRI cells and the newly differentiated VRI cells are reinfected by SARS-CoV-2, leading to a further increase in the number of infected VRI cells.

To determine why number of ciliated cells decreases and that of VRI cells increases following SARS-CoV-2 infection, we investigated apoptosis markers in VRI and ciliated cells over different time points. In ciliated cells, the pro-apoptotic gene BAX and the executioner caspase CASP3 showed a time-dependent increase in expression following infection. However, in VRI cells, the expression of these two genes decreased up to 2 dpi, while the anti-apoptotic gene BCL2 increased (Figure 5E). Additionally, the anti-apoptotic gene PEBP4, which is also involved in lipid metabolism, showed increased expression in VRI_stage2 (Figure 2C). This indicates that apoptosis is suppressed in VRI cells until the peak of infection, likely allowing VRI cells to contribute to the SARS-CoV-2 life cycle by evading cell death. In contrast, ciliated cells initially increased in number after infection but then decreased, which can be attributed to sustained apoptosis.

## 4 Discussion

Here, we investigated the early dynamics of human airway epithelial infection by SARS-CoV-2 and identified an uncharacterized cell population, VRI cells. These VRI cells, positioned between basal and ciliated cells during differentiation, play a critical role in viral replication due to their high viral RNA content. Notably, we identified a subset, VRI_stage2, which only appears post-infection and displays nearly 100% infection (Figure 2B; Supplementary Figure S4). Our analysis shows that VRI cells are intermediate cells transitioning from basal to ciliated cells, and SARS-CoV-2 exploits these cells for replication, rather than fully differentiated ciliated cells, which were traditionally seen as the virus’s primary targets. Our pseudo-time trajectory analysis supports the idea that SARS-CoV-2 exploits these intermediate cells for replication (Figure 2E), likely due to their dynamic state of differentiation. The significantly higher proportion of infected VRI cells compared to other cell types may explain why some individuals experience prolonged or severe infections. Continuous viral replication in VRI cells could contribute to sustained viral shedding, as evidenced by the persistence of infected VRI cells even at 28 dpi (Figures 3A,B). These findings suggest that targeting VRI cells could offer a potential therapeutic strategy to reduce viral load and limit disease progression.

Building on this, VRI cells warrant further investigation into their identity and function. While they act as a transitional population differentiating from basal to ciliated cells under mock conditions, they emerge as a distinct cell cluster post-SARS-CoV-2 infection, raising the possibility of a novel cell type induced by viral infection. Notably, VRI_stage2, which arises only post-infection and exhibits nearly 100% infection, highlights this distinct identity (Supplementary Figure S4). Key markers such as ZBTB10, DUSP8, and HIST1H2BG, predominantly expressed in VRI_stage2, suggest specialized roles during infection beyond the transitional differentiation observed in uninfected conditions (Supplementary Table S3). These markers, particularly ZBTB10, provide a valuable tool for isolating VRI cells in transwell systems, enabling cytological studies to clarify their structural and functional characteristics. Such experiments will be instrumental in determining whether VRI cells represent a new cell type or an intermediate state with uniquely adapted functions during infection, further elucidating their role in viral replication and pathogenesis.

Our findings also align with clinical observations from COVID-19 patients. Clinical nasal swabs from COVID-19 patients revealed regions populated by multiple secretory and intermediate cell subsets, similar to the VRI populations we identified, which were largely absent in control participants (Ziegler et al., 2021). The accumulation of these intermediate cells may contribute to the impaired mucociliary clearance seen in severe COVID-19 cases, as the virus disrupts normal epithelial cell differentiation and function.

One of the key findings of our study is that VRI cells exhibit altered gene expression patterns that may enhance viral replication. Our GO enrichment analysis revealed an upregulation of genes associated with cilia assembly after infection (Figure 5A), suggesting that SARS-CoV-2 promotes ciliary activity during the early stages of infection. Importantly, VRI_stage3 showed particularly strong enrichment for cilia-related processes, including cilium organization and microtubule-based movement (Figure 2D). This aligns with the trajectory analysis, which shows the transition from VRI_stage1 to VRI_stage2, and eventually to VRI_stage3 (Figure 2E; Supplementary Figure S2), where infected cells likely begin cilia formation. Infected cell also showed significant enrichment in fluid regulation pathways, while gland development pathways were observed in bystander cells (Figure 5A). This process contributes to a favorable environment for viral dissemination by improving mucus transportation. This suggests that cilia formation, fluid dynamics, and gland development work together to enhanced viral spread by optimizing the environment for viral propagation.(Wu et al., 2023; Koca et al., 2021). Additionally, the high expression of ACE2 in cilia makes these cells more susceptible to SARS-CoV-2 entry during early infection (Robinot et al., 2021; Jia et al., 2005; Kawasumi et al., 2022; Lee et al., 2020; Buqaileh et al., 2021). This combination of cilia formation, fluid regulation, and high ACE2 expression in VRI cells creates an optimal environment for viral entry and propagation, allowing the virus to spread more efficiently within the airway. Fluid regulation.

However, this model contrasts with other studies suggesting SARS-CoV-2 eventually causes the loss of cilia, which might facilitate the virus’s deeper penetration into the lung parenchyma (Ziegler et al., 2021; Ahn et al., 2021; Robinot et al., 2021; Bridges et al., 2022). In our analysis, a decrease in ciliated cells was observed starting at 3 dpi (Figure 3A), aligning with previous studies that reported ciliated cell depletion at around 4 dpi (Robinot et al., 2021). This temporal pattern suggests that SARS-CoV-2 initially upregulates cilia formation and movement to maximize its spread, but as the infection progresses, the virus may induce ciliary depletion, potentially aiding in its deeper invasion into the respiratory tract. Specifically, we found that SARS-CoV-2 induces microvilli formation in VRI cells, driven by the upregulation of key genes such as EZR and VIL1, which is consistent with prior research showing that microvilli enhance viral budding and release (Wu et al., 2023; Bretscher and Weber, 1979; Gaeta et al., 2021) (Figure 5B). When combined with the observed increased cilia formation and movement during the infection, as well as fluid regulation pathways enriched in infected cells, these results suggest that SARS-CoV-2 utilizes a multi-faceted strategy: microvilli for efficient viral release and enhanced ciliary activity for spreading the virus across surrounding cells, and fluid regulation to create a favorable environment for viral dissemination within the host.

In addition to structural adaptations, SARS-CoV-2 also actively modulates the host’s immune response to maintain a replicative advantage. Our analysis of immune-related genes in VRI cells underscores the intricate interaction between SARS-CoV-2 and the host’s antiviral defenses. VRI_stage2, the most heavily infected subset, displayed robust interferon and inflammatory responses, including elevated expression of IFNL1, IFNB1, and ISGs such as OAS1, MX1, and ISG15 (Figure 4A). Despite this immune activation, the expression of certain ISGs, including IFIT1 and ISG15, was lower in VRI_stage2 compared to VRI_stage1 (Figures 4A,B), suggesting that SARS-CoV-2 may employ immune evasion mechanisms to dampen specific antiviral responses. This differential ISG activation across VRI subtypes implies that the virus may fine-tune the host immune response at different stages of cell differentiation, optimizing conditions for viral replication.

However, while these immune interactions highlight the virus’s ability to sustain infection, they do not fully explain the observed changes in cell populations, particularly the increase in VRI cells. To further explore this, we performed a cell-cell interaction analysis, revealing that VRI cells send NAMPT and COL1A1 signals to both basal and ciliated cells. NAMPT signaling is well-known for its role in regulating immune responses and activating inflammatory processes, acting similarly to cytokines and DAMPs in SARS-CoV-2 infection (Audrito et al., 2020). This suggests that VRI cells engage in immune modulation. COL1A1, on the other hand, is crucial for extracellular matrix remodeling by interacting with integrins, particularly in tissue repair and fibrosis. By triggering the Akt/PI3K pathway through integrins such as ITGA1/ITGB1, it promotes cell survival, growth, and migration (Devos et al., 2023). These pathways likely support tissue remodeling and may contribute to fibrosis during infection. The presence of these signals underscores the potential of VRI cells to not only sustain viral replication but also drive pathological changes in the infected tissue.

It is noteworthy that, in addition to the NAMPT and COL1A1 signaling pathways, VRI cells also send EPHA, EPHB, and VEGFA signals specifically to basal cells (Figure 5C). Eph-ephrin signaling is known to have direct effects on cell differentiation within tissues (Wilkinson, 2014), while VEGFA stimulates the proliferation and growth of basal cells through a VEGFR2-dependent pathway. These signaling pathways are closely associated with the MAPK pathway, a key regulator of cell growth, differentiation, and survival (Perez White and Getsios, 2014; Doanes et al., 1999; Sun et al., 2015). Our GO enrichment analysis in this study showed the significant upregulation of MAPK pathways in basal cells following SARS-CoV-2 infection; therefore, it confirms our cell-cell interaction analysis result (Figure 5D). This correlation would indicate that the MAPK pathway has a significant role in promoting basal cell differentiation into VRI cells. By manipulating this pathway, the virus likely secures a reliable source of target cells, enhancing its capacity for replication in the host. These dynamic highlights the critical importance of basal cell differentiation in sustaining the ongoing infection process and facilitating the regeneration and expansion of VRI cells, further supporting viral replication.

Notably, the Ephrin-Eph signaling pathway, which VRI cells utilize to signal basal cells, has been extensively studied for its role in viral infections. The review “Ephrin-Eph signaling usage by a variety of viruses” emphasizes how various viruses, including Hendra and Nipah viruses, exploit this pathway to mediate their entry into target cells and modulate cell adhesion to establish infection (de Boer et al., 2020). Since SARS-CoV-2 also uses Ephrin-Eph signaling to connect VRI to basal cells, the same might be true for how the virus manipulates the pathway. Even though speculative, it is possible that SARS-CoV-2 could exploit Ephrin-Eph signaling to modify the host cellular environment and, therefore, mediate its dissemination through alterations in cell-cell interactions.

Finally, our study provides new insights into how SARS-CoV-2 manipulates apoptosis pathways to enhance its survival within host cells. we observed that SARS-CoV-2 infection led to the upregulation of BCL2 in VRI cells, which is associated with the suppression of apoptosis in these cells (Figure 5E). This contrasts with the response in ciliated cells, where such an anti-apoptotic effect was not observed. The upregulation of BCL2 suggests that SARS-CoV-2 may be employing a strategy similar to other viruses that use BCL2 to extend the survival of their host cells (Kvansakul et al., 2017; Barber, 2001). By upregulating BCL2, SARS-CoV-2 likely extends the survival of VRI cells, maintaining a stable environment for ongoing viral replication.

Despite the significant findings of this study, several limitations should be noted. First, our *in vitro* models using human nasal and tracheal epithelial cells may not fully capture the complexity of *in vivo* environments, particularly immune responses and interactions with other cell types, limiting direct extrapolation to clinical settings. Further experimental validation *in vivo* will be essential to confirm the role of VRI cells and associated signaling pathways, providing a more comprehensive understanding of their biological significance and therapeutic potential. Second, combining publicly available single-cell RNA sequencing datasets might introduce variability due to differences in experimental conditions and data processing methods. Although we applied rigorous integration methods, including batch effect correction and subsampling, to minimize biases and ensure robust analyses, this potential limitation should be acknowledged. Third, while the study focused on the early stages of infection (1–28 days), it did not investigate the long-term effects or potential post-infection sequelae, essential for understanding chronic infections or long COVID. Moreover, the ALI culture system employed, though adequate for mimicking epithelial cells under certain conditions, raises questions about the generalizability of the results across different populations or environmental contexts. Finally, while the study highlights the differentiation of VRI cells as a transitional phase from basal to ciliated cells, further investigations are needed to elucidate the underlying mechanisms and regulatory factors guiding this differentiation process.

**Figure 6:**
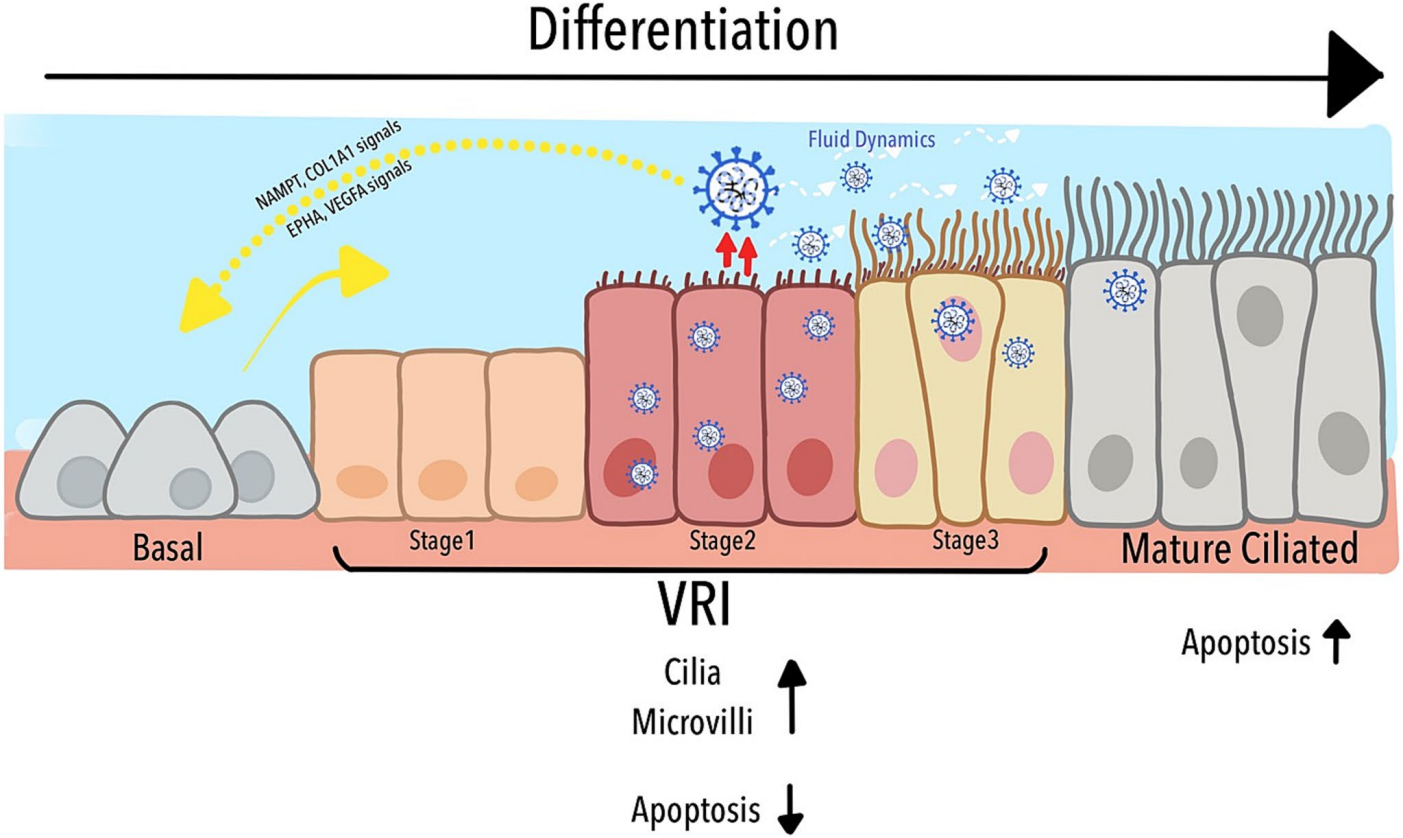
Schematic representation of VRI cell differentiation and role in SARS-CoV-2 infection.

As depicted in Figure 6, our study identified a previously uncharacterized population of Virus-Rich Intermediate (VRI) cells, positioned between basal and mature ciliated cells in the differentiation pathway of the airway epithelium. These VRI cells play a critical role in SARS-CoV-2 replication, acting as a transitional hub where the virus manipulates both cellular structure and function. The figure illustrates how VRI cells progress through distinct stages (VRI_stage1 to VRI_stage3) during infection, with VRI_stage2 exhibiting nearly complete viral infection. SARS-CoV-2 exploits the differentiation process by inducing cilia assembly, microvilli formation, and fluid regulation, all of which enhance viral replication and dissemination. Furthermore, while mature ciliated cells undergo apoptosis, facilitating the virus’s deeper invasion into the respiratory tract, VRI cells suppress apoptosis, maintaining an environment that supports continuous viral replication. The cell-cell signaling pathways between VRI cells and basal cells, such as NAMPT/COL1A1 and EPHA/VEGFA, contribute to tissue remodeling and immune modulation, further aiding the virus’s ability to thrive within the host. Together, these processes create a dynamic environment that the virus manipulates to optimize its replication and survival within the airway epithelium.

In conclusion, our study reveals that SARS-CoV-2 intricately modulates the host cell population and morphology during early infection to optimize its replication and spread. The identification of VRI cells as a key player in this process highlights the virus’s ability to manipulate host cell differentiation, immune responses, and apoptosis to its advantage. These findings provide a deeper understanding of SARS-CoV-2 pathogenesis and suggest new potential targets for therapeutic intervention. Targeting the pathways that promote differentiation of VRI cells, viral replication, and immune evasion may offer new strategies for controlling SARS-CoV-2 infection and mitigating its impact on the respiratory epithelium.

## Data Availability

Publicly available datasets were analyzed in this study. This data can be found here: (GSE166766) https://www.ncbi.nlm.nih.gov/geo/query/acc.cgi?acc=GSE166766, (GSE157526) https://www.ncbi.nlm.nih.gov/geo/query/acc.cgi?acc=GSE157526, (GSE182475) https://www.ncbi.nlm.nih.gov/geo/query/acc.cgi?acc=GSE182475.
