# Supplementary figures and images for: Identification of virus-rich intermediate cells as crucial players in SARS-CoV-2 infection and differentiation dynamics of human airway epithelium
Source: Front Microbiol. 2024 Dec 13;15:1507852. doi: 10.3389/fmicb.2024.1507852 (PMC11681626; doi:10.3389/fmicb.2024.1507852)

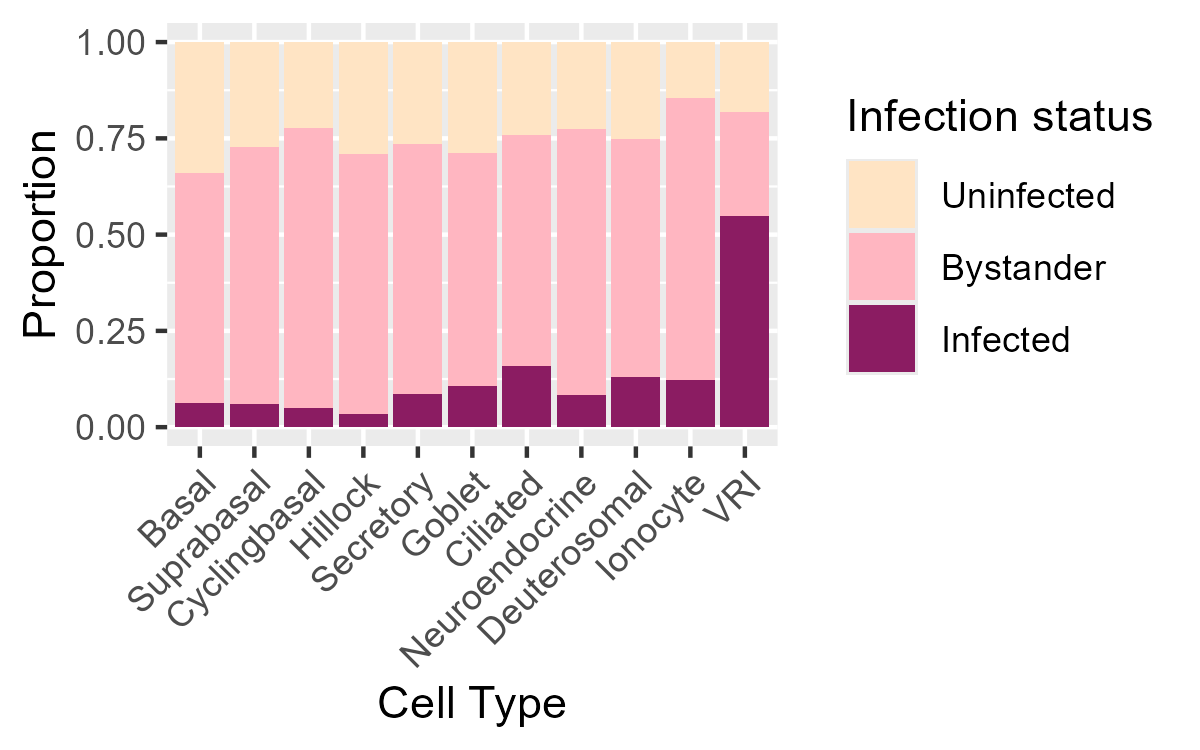

Supplement: Supplementary file 1 [file Image_1.PNG]

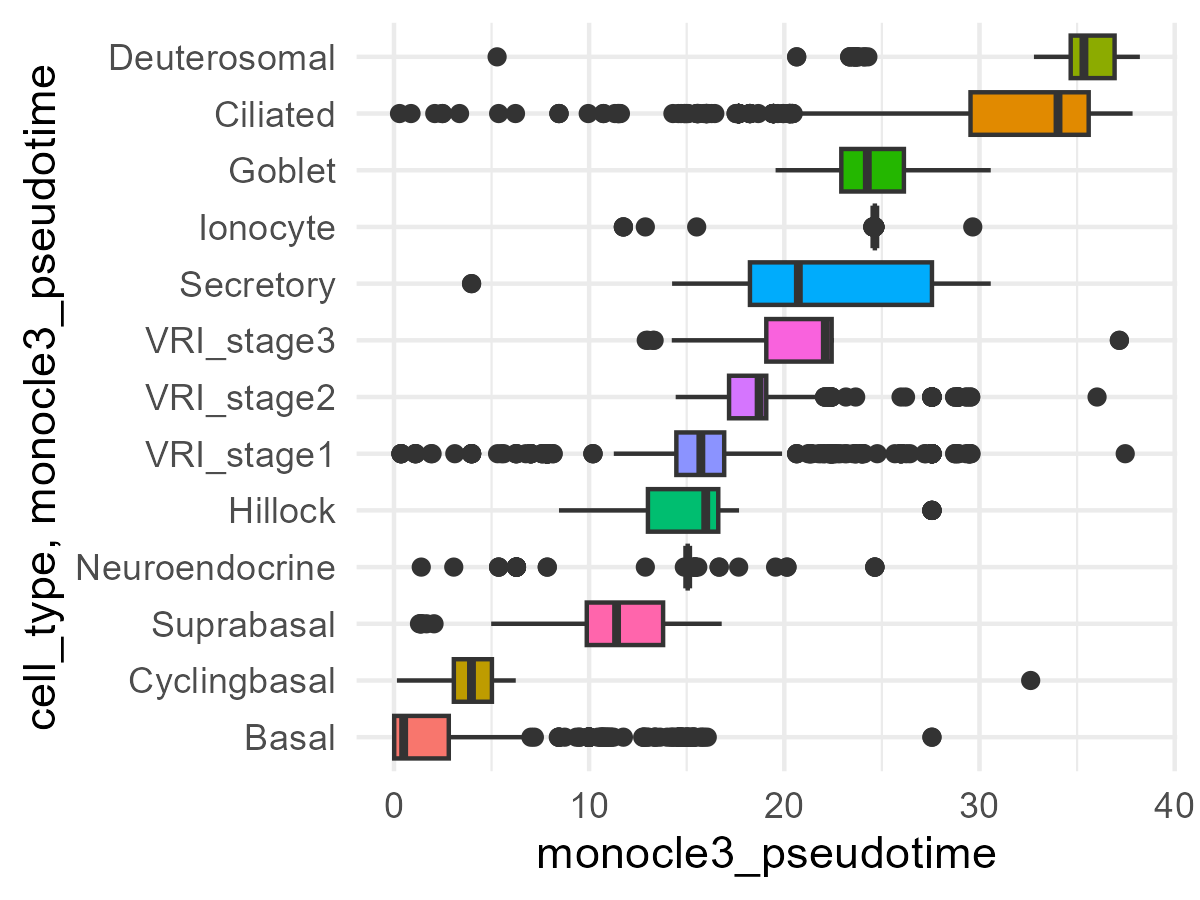

Supplement: Supplementary file 2 [file Image_2.PNG]

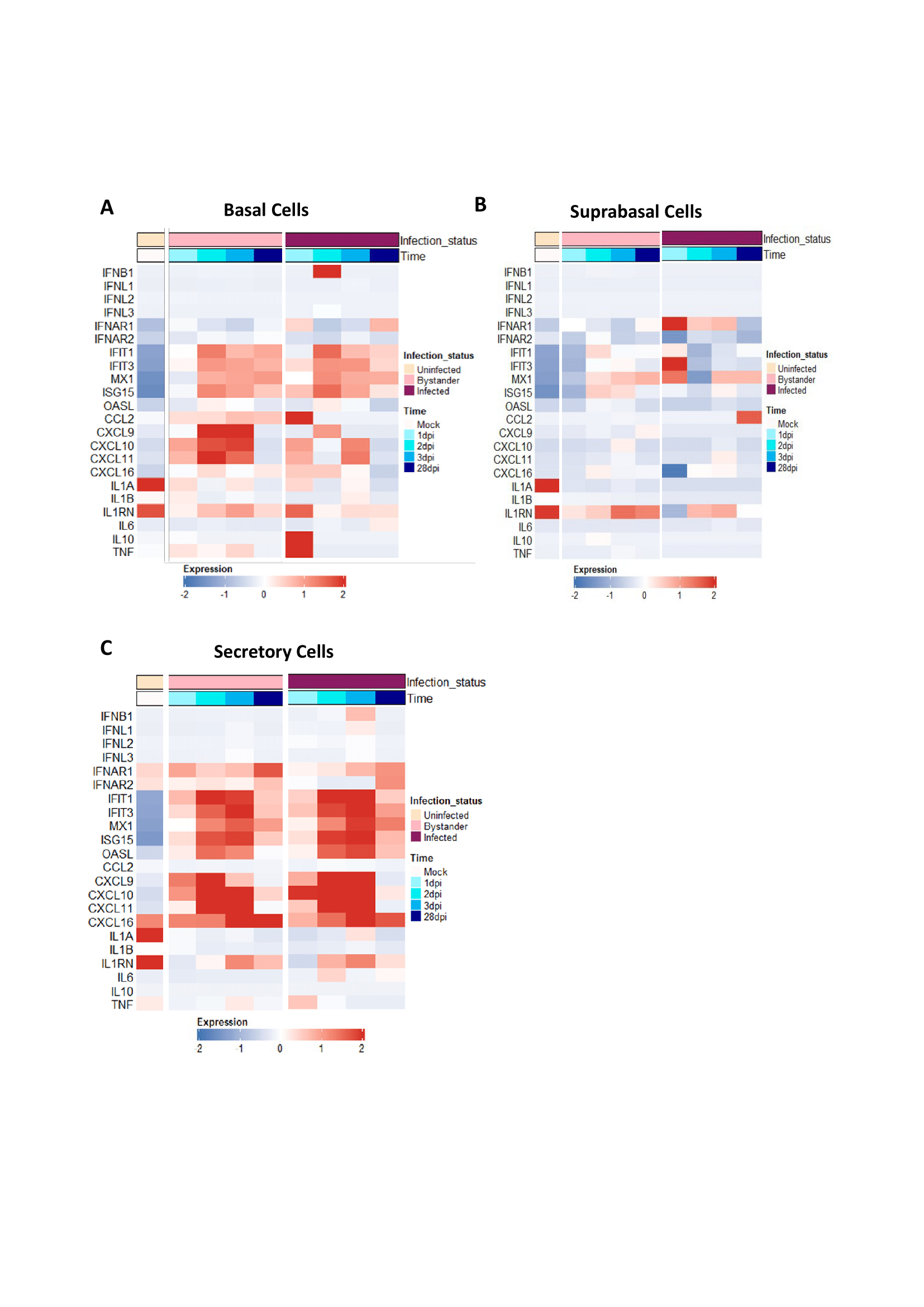

Supplement: Supplementary file 3 [file Image_3.PNG]

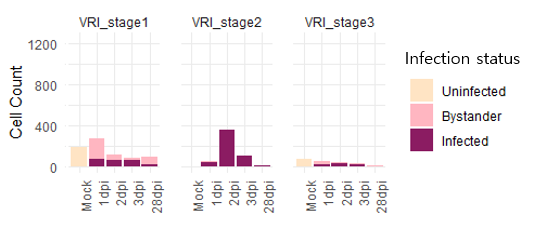

Supplement: Supplementary file 4 [file Image_4.PNG]
