# Supplementary material for: Identification of virus-rich intermediate cells as crucial players in SARS-CoV-2 infection and differentiation dynamics of human airway epithelium
Source: Front Microbiol. 2024 Dec 13;15:1507852. doi: 10.3389/fmicb.2024.1507852 (PMC11681626; doi:10.3389/fmicb.2024.1507852)
Supplement: Supplementary file 5 [file Table_1.DOCX]

Supplementary Material

# Supplementary Tables

**Supplementary Table 1: Subsampling of cells from each dataset for scRNA-seq analysis.**

| **Time** | **Ravindra et al.** | **Fiege et al.** | **Gamage et al.** | **Total cell number** |
| --- | --- | --- | --- | --- |
| **Mock** | 1700 | 1700 | 1700 | 5,100 |
| **1dpi** | 1700 | 1700 | 0 | 3,400 |
| **2dpi** | 1700 | 1700 | 0 | 3,400 |
| **3dpi** | 1700 | 0 | 1700 | 3,400 |
| **28dpi** | 0 | 0 | 3400 | 3,400 |

**Supplementary Table 2: airway epithelial cell marker genes used for scRNA-seq cluster annotation**

| **Cell types** | **Marker gene** |
| --- | --- |
| **Basal** | KRT5, TP63,PDPN,NGFR,LGALS1,ITGA6, ITGB4, LAMA3, LAMB3, KRT15, S100A2, NPPC, BCAM, DST |
| **Cycling Basal** | MKI67, TOP2A |
| **Suprabasal** | KRT19, NOTCH3 |
| **Hillock** | KRT13, KRT4 |
| **Secretory** | SCGB1A1, MUC5AC, MUC5B, TFF3, SCGB3A1, BPIFB1, MSMB, SLPI, WFDC2,KRT7, CXCL17, F3, AQP5, CP,EPIFA1 |
| **Goblet** | MUC5AC high |
| **Ciliated** | FOXJ1, PIFO, TPPP3, SNTN, FAM183A, LRRIQ1, DNAH12, C20orf85, CAPS, TUBB4B, DNAH5, TSPAN1,DLEC1, DNAH11, CFAP43 |
| **Neuroendocrine** | CHGA, ASCL1, INSM1, HOXB5, DLEC1, DNAH11, CFAP43 |
| **Ionocyte** | FOXI1, CFTR, ASCL3, RARRES2 |
| **Deuterosomal** | FOXN4, CDC20B,DEUP1, FOXJ1,CCNO, CDC20B, HES6 |

**Supplementary Table 3: Cell type-specific marker genes identified using the FindAllMarkers function in Seurat.**

# Supplementary Figures legend

# Supplementary Figure 1. Proportions of uninfected, bystander, and infected cells across various epithelial cell types, including Virus-Rich Intermediate (VRI) cells. Cell types are labeled on the x-axis, and the y-axis represents the proportion of cells in each infection category.

# Supplementary Figure 2. Distribution of Epithelial Cell Subtypes Along Pseudotime. Boxplot showing the distribution of epithelial cell subtypes along pseudotime, including Virus-Rich Intermediate (VRI) cells at different stages. Colors correspond to different subtypes.

**Supplementary Figure 3. Basal, intermediate, and secretory cells exhibit distinct immune and inflammatory gene expression profiles following viral infection.** (A-C) Heatmaps depicting the expression levels of key inflammatory and immune response genes across time and infection status in different epithelial cell populations: (A) Basal cells, (B) Intermediate cells, and (C) Secretory cells. Each column represents a pseudobulk average of all cells from a single cell type and infection status (uninfected, bystander, infected) at multiple time points (mock, 1dpi, 2dpi, 3dpi, 28dpi). The color scale indicates z-score-transformed expression levels, with red representing higher expression and blue representing lower expression.

**Supplement Figure 4: Distribution of infection status across VRI_stage cell populations.** (A) Bar plots depicting the number of cells classified as uninfected, bystander, or infected across the three VRI stages: VRI_stage1, VRI_stage2, and VRI_stage3. Each bar represents the total cell count for each infection status at different time points (0h, 24h, 72h). Infected cells are shown in purple, bystander cells in light pink, and uninfected cells in gray.
